# Anesthesia considerations to reduce motion and atelectasis during advanced guided bronchoscopy

**DOI:** 10.1186/s12890-021-01584-6

**Published:** 2021-07-17

**Authors:** Michael A. Pritchett, Kelvin Lau, Scott Skibo, Karen A. Phillips, Krish Bhadra

**Affiliations:** 1 Chest Center of the Carolinas at First Health, President of the Society for Advanced Bronchoscopy, FirstHealth of the Carolinas and Pinehurst Medical Clinic, 205 Page Road, Pinehurst, NC 28374 USA; 2 Thoracic Surgery, St. Bartholomew’s Hospital, West Smithfield, London, EC1A 7BE UK; 3 Interventional Thoracic Oncology, Pulmonary Critical Care, Haywood Regional Medical Center (A Duke LifePoint Hospital), 262 Leroy George Drive, Clyde, NC 28721 USA; 4 Anesthesiologist and Intensivist, Medtronic, 2101 Faraday Avenue, Carlsbad, CA 92008 USA; 5 Interventional Pulmonology, CHI Memorial Rees Skillern Cancer Institute, 725 Glenwood Dr E-500, Chattanooga, TN 37401 USA

**Keywords:** Atelectasis, General anesthesia, Electromagnetic navigation bronchoscopy, Radial endobronchial ultrasound, Image-guided bronchoscopy, Computed tomography, Divergence, Lung cancer

## Abstract

Partnership between anesthesia providers and proceduralists is essential to ensure patient safety and optimize outcomes. A renewed importance of this axiom has emerged in advanced bronchoscopy and interventional pulmonology. While anesthesia-induced atelectasis is common, it is not typically clinically significant. Advanced guided bronchoscopic biopsy is an exception in which anesthesia protocols substantially impact outcomes. Procedure success depends on careful ventilation to avoid excessive motion, reduce distortion causing computed tomography (CT)-to-body-divergence, stabilize dependent areas, and optimize breath-hold maneuvers to prevent atelectasis. Herein are anesthesia recommendations during guided bronchoscopy. An FiO_2_ of 0.6 to 0.8 is recommended for pre-oxygenation, maintained at the lowest tolerable level for the entire the procedure. Expeditious intubation (not rapid-sequence) with a larger endotracheal tube and non-depolarizing muscle relaxants are preferred. Positive end-expiratory pressure (PEEP) of up to 10–12 cm H_2_O and increased tidal volumes help to maintain optimal lung inflation, if tolerated by the patient as determined during recruitment. A breath-hold is required to reduce motion artifact during intraprocedural imaging (e.g., cone-beam CT, digital tomosynthesis), timed at the end of a normal tidal breath (peak inspiration) and held until pressures equilibrate and the imaging cycle is complete. Use of the adjustable pressure-limiting valve is critical to maintain the desired PEEP and reduce movement during breath-hold maneuvers. These measures will reduce atelectasis and CT-to-body divergence, minimize motion artifact, and provide clearer, more accurate images during guided bronchoscopy. Following these recommendations will facilitate a successful lung biopsy, potentially accelerating the time to treatment by avoiding additional biopsies. Application of these methods should be at the discretion of the anesthesiologist and the proceduralist; best medical judgement should be used in all cases to ensure the safety of the patient.

**Supplementary Information:**

The online version contains supplementary material available at 10.1186/s12890-021-01584-6.

## Background

Advanced guided bronchoscopic biopsy is a minimally invasive method to diagnose suspicious lung lesions [1, 2]. Guidance modalities include not only intraprocedural image-guided methods (e.g., augmented fluoroscopy, tomosynthesis-based fluoroscopic navigation), but also electromagnetic navigation bronchoscopy (ENB), electromagnetic-based and fiber-optic shape-sensing robotic bronchoscopy, virtual bronchoscopic navigation (VBN), radial endobronchial ultrasound (EBUS), and cone-beam computed tomography (CBCT) [1, 2]. Guided bronchoscopy has a lower risk of complications than transthoracic biopsy [3, 4], and is particularly useful for small lesions in the periphery of the lung that cannot be reached by traditional bronchoscopy [5]. Early diagnosis and treatment of malignant lung lesions can substantially increase survival rates [6, 7].

Despite these advantages, the diagnostic yield of all guided bronchoscopy systems has historically been limited by a phenomenon called computed tomography (CT)-to-body divergence [8]. This effect refers to a mismatch between the preprocedure CT-based virtual map used to plan the navigation route to the lung lesion and the actual dynamic lung anatomy during bronchoscopy. This divergence can be caused by a number of factors [8] and has been addressed, in part, by advanced bronchoscopy systems that provide real-time visualization and/or positional correction during the bronchoscopy procedure [9–18]. However, anesthesia-induced atelectasis remains a challenge that can obscure lesion visibility and cause inaccurate localization, even with intraprocedural positional correction.

While it has long been known that nearly all patients experience atelectasis within minutes after general anesthesia induction [19–23], atelectasis during bronchoscopy and its impact on outcomes has been underappreciated until recently. Because atelectasis is not visible on standard fluoroscopy [24, 25], its prevalence during bronchoscopy was not realized until the use of CBCT became more common. Using CBCT, Casal et al. were the first to report atelectasis in dependent areas in 40% of peripheral lung biopsies, completely obscuring the target in 20% of cases [24]. Avasarala et al. reported atelectasis in 75% of patients, obscuring the lesion in 38% [26]. In the first prospective study specifically designed to assess atelectasis during bronchoscopy (I-LOCATE), Sagar et al. reported that 89% of patients had atelectasis in at least one bronchial segment with a prevalence greater than 50% in dependent lower lobe segments. Atelectasis was observed within 30 min of anesthesia induction. Increased body mass index and time to atelectasis assessment were significant predictors of atelectasis risk [25]. General anesthesia was used with a laryngeal mask airway (LMA) and neuromuscular blocking agents, and most patients were ventilated with 100% fraction of inspired oxygen (FiO_2_) and zero to minimal PEEP [25].

**Fig. 1:**
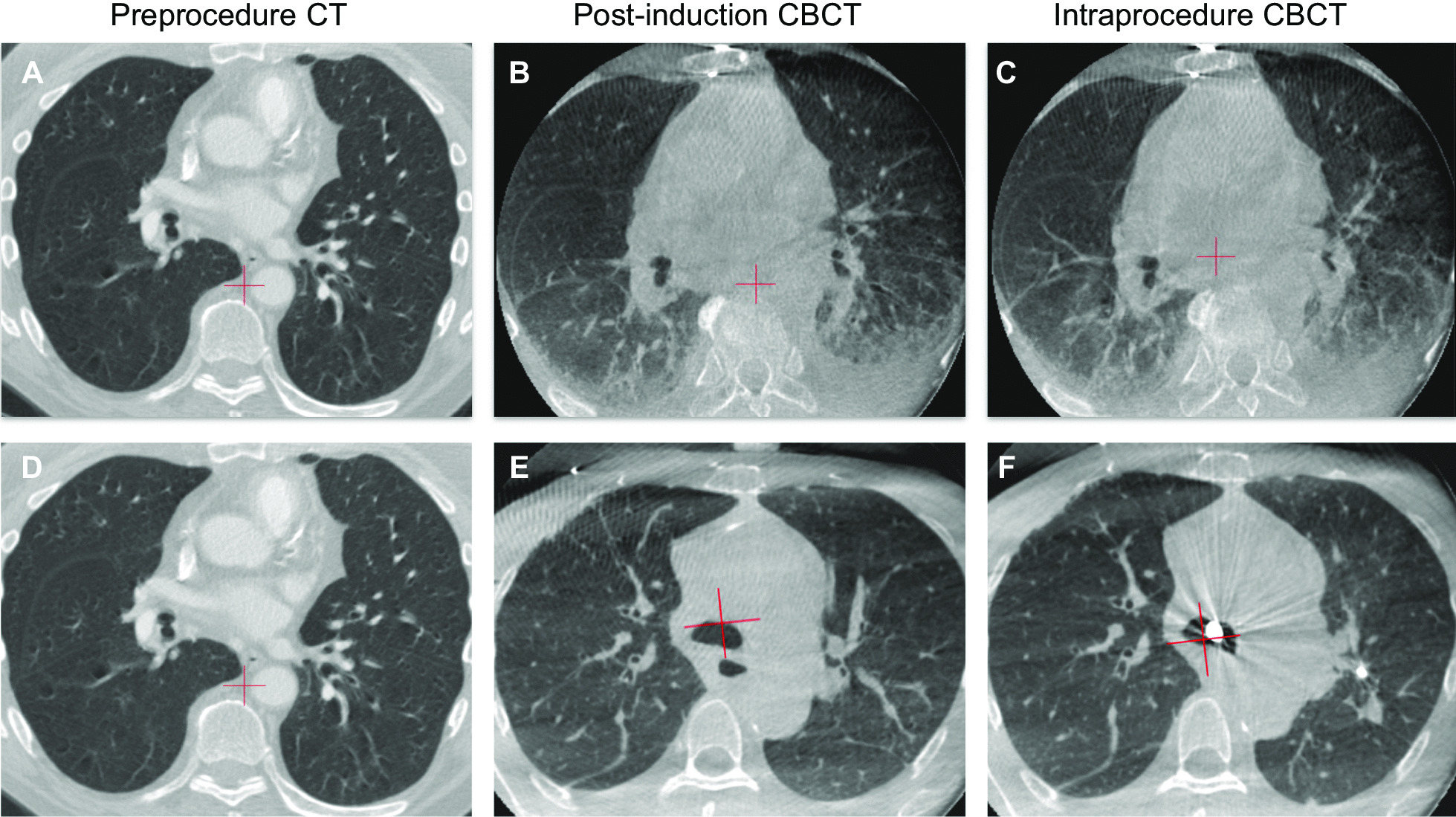
**A**–**C** Computed tomography (CT) and cone-beam CT (CBCT) scans during image-guided bronchoscopy without an optimized ventilation protocol. Significant atelectasis and ghosting artifact were observed. **D**–**F** CT and CBCT scans from a different patient using a ventilation protocol designed to prevent atelectasis

Atelectasis can have a significant impact on the success of guided bronchoscopy. Atelectasis decreases the visibility of lung lesions on imaging and changes the conformation of the airways (Fig. 1), exacerbating any mismatch between the virtual lung map and the patient’s anatomy. Atelectasis reduces the distance between the lesion and the pleura, increasing the risk of pneumothorax from instrumentation. Because of its increased density compared to aerated lung, atelectasis can also create false positive findings on radial EBUS that mimic a lung lesion [25]. The result is inaccurate localization and reduced diagnostic yield [27]. Significant changes in inflation pressure are required to open atelectatic lung units. Because recruitment maneuvers to temporarily increase PEEP and tidal volumes often fail to resolve the atelectasis once it has appeared [24], it is important to prevent atelectasis from occurring and maintain optimal conditions throughout the bronchoscopic navigation and biopsy procedures.

## Literature review

A systematic literature search of MEDLINE and Embase was conducted for papers evaluating the impact of anesthesia methods on outcomes during advanced peripheral bronchoscopy (ENB, radial EBUS, VBN, CBCT, augmented fluoroscopy, robotic bronchoscopy). Detailed methods are provided in Additional file 1: Figure S1, Additional file 1: Table S1 and Additional file 1: Table S2. After abstract and full-text review, there were 11 papers (8 original studies and 3 meta-analyses) that specifically evaluated the impact of the anesthesia method on atelectasis, safety, or diagnostic yield during advanced bronchoscopy [4, 27–36]. Bowling et al. compared ENB using general anesthesia versus intravenous sedation and observed diagnostic yields of 70% and 78%, respectively (*P* = NS) [29]. As will be described in greater detail below, Bhadra et al. 2021 observed less atelectasis and a trend toward greater diagnostic yield using an optimized ventilation protocol compared to conventional ventilation [28]. Minami et al. (2017) reported that fentanyl and midazolam sedation was useful for peripheral bronchoscopy [32]. Webb et al. (2020) reported that jet ventilation was associated with reduced target displacement and increased diagnostic yield compared to traditional ventilation during ENB [35]. General anesthesia and conscious sedation were used with approximately equal frequency across studies in a 2020 meta-analysis with no significant differences in diagnostic yield [4], consistent with results from the AQuIRE registry [33], a 2015 meta-analysis [36], and the NAVIGATE study [30]. One 2014 meta-analysis observed significantly higher diagnostic yield with general anesthesia use compared to conscious sedation [31] and the NAVIGATE study found a significant effect of general anesthesia versus moderate sedation on complication rates [34]. Finally, Tanner et al. found no significant difference in diagnostic yield using moderate versus deep sedation using standard bronchoscopy or radial EBUS [27].

However, with one exception [28], the studies described above used navigation bronchoscopy systems without intraprocedural advanced imaging. Newer systems such as tomosynthesis-based fluoroscopic navigation [10] and CT augmented fluoroscopy [28] require a breath hold to minimize lung movement during intraprocedural imaging and location adjustments. Furthermore, CT-to-body divergence and atelectasis are likely strong contributors to the sub-optimal diagnostic yield seen with earlier generations of navigation bronchoscopy technology in large multicenter studies [30].

## Ventilation recommendations

The following is a recommended protocol for ventilation during advanced guided bronchoscopic biopsy (Table 1). These recommendations were derived from the anesthesia literature regarding the principles of minimizing atelectasis during positive pressure ventilation [37–42], the authors’ published work [28], and the authors’ combined experience of over 1000 CBCT navigation cases assessing the presences of atelectasis and CT-to-body divergence. While the use of higher PEEP and lower FiO_2_ during induction may contradict traditional anesthesiology practices, they are important for guided bronchoscopy where the success of the biopsy is highly dependent upon stabilizing dependent areas of the lung during intraprocedural imaging, particularly in obese patients and those with posterior or lower lobe lesions [43, 44]. These techniques will help to reduce movement and minimize atelectasis, thereby increasing the chances of a successful biopsy and potentially accelerating the time to treatment by reducing the need for repeat biopsy procedures. These methods should be used at the discretion of the anesthesiologist and the proceduralist based on the individual risk factors of each patient. Standard monitoring procedures should be followed according to the American Society of Anesthesiologists (ASA) recommendations.

**Table 1:** Anesthesia for advanced guided bronchoscopy

	Step	Considerations	Recommendations
1	Preprocedure	Recruit lung volume, assess tolerance to higher PEEP, and prevent atelectasis	Perform incentive spirometry
2	Preoxygenation	Avoid absorption atelectasis	Modest FiO_2_ (0.6 to 0.8) as tolerated
3	Anesthesia type	Need for a completely motionless patient	TIVA with propofol and muscle paralysis
4	Intubation	Enable gas passage past the bronchoscope with the least increase in circuit pressure	Use a larger endotracheal tube (usually ≥ 8.5, but as guided by patient anatomy)
		Minimize atelectasis by avoiding traditional rapid-sequence intubation (i.e., avoid FiO_2_ of 1.0 and Suxamethonium)	Perform an expeditious intubation using non-depolarizing muscle relaxants
5	Post-intubation	Reverse any induction-related atelectasis and assess hemodynamic stability during higher PEEP	Conduct up to 4 recruitment maneuvers as tolerated
			Maintain FiO_2_ at the lowest tolerable level
		Maintain optimal lung inflation	PEEP of up to 10–12 cm H_2_O for upper lobe biopsies, consider higher PEEP for lower lobe lesions or obese patients
			An increase in tidal volumes may be considered
6	Breath-hold: timing	Reduce motion artifact	Breath-hold at peak inspiration (end of a normal tidal breath)
	Breath-hold: pressure	Maintain a constant circuit pressure and PEEP and reduce diaphragmatic movement	Manually adjust APL valve to maintain circuit pressure at desired PEEP level
	Breath-hold: duration	To minimize lung movement during imaging, allow time for pressure to equilibrate	Maintain breath-hold for 5–10 s before beginning imaging sweep
7	Biopsy	Ensure consistent settings between imaging and biopsy	Maintain settings at the same levels as Step 6
8	Post-procedure	Exclude pneumothorax and assess any residual atelectasis	Routine reversal and post-procedure methods. Perform chest X-ray

*APL* adjustable pressure-limiting valve, *FiO*_*2*_ fraction of inspired oxygen, *PEEP* positive end-expiratory pressure, *TIVA* total intravenous anesthesia

### Preprocedure incentive spirometry

Preprocedure incentive spirometry has been recommended to recruit lung volume and prevent atelectasis [45]. The incentive spirometry maximum value will also allow the anesthesiologist to anticipate any risks of the higher intraprocedural tidal volumes recommended below. This is especially important for patients at particular risk of intraprocedural atelectasis, such as obese patients or those with lesions in the dependent areas of the lung.

### Preoxygenation

The benefits of pre-oxygenation prior to anesthetic induction and tracheal intubation are widely accepted. However, nitrogen washout during pre-oxygenation promotes loss of gas from the lung to the bloodstream, resulting in alveolar collapse and absorption atelectasis. Gas absorption accelerates airway collapse by a combination of decreased functional residual capacity and compression atelectasis [46]. Therefore, the use of 100% oxygen during induction and anesthesia maintenance is a major cause of atelectasis [41]. Given the risk of absorption atelectasis and lung injury, the lowest tolerable FiO_2_ is recommended for preoxygenation [46] as guided by oxygen saturation. Edmark et al. demonstrated that in most patients, an FiO_2_ of 0.8 to 0.6 was associated with little to no atelectasis compared to an FiO_2_ of 1.0 [37]. Inspired oxygen content should also be kept as low as tolerated during the procedure to minimize absorption atelectasis.

If it is not possible to decrease the FiO_2_ to less than 1.0 during pre-induction, the FiO_2_ should be kept at the lowest tolerable level immediately after the endotracheal tube (ETT) cuff is inflated, and recruitment maneuvers should be performed with the lowered FiO_2_. This lowered FiO_2_ should be maintained for the remainder of the procedure, including during the biopsy, as the patient’s oxygen saturation permits.

### Anesthesia type and intubation

The need for a completely motionless patient during guided bronchoscopy necessitates general anesthesia, paralysis, and intubation. The largest ETT size feasible based on patient anatomy will enable gas passage around the bronchoscope (which ranges in diameter from 5.9 to 6.3 mm) with the least increase in circuit pressure [47]. An ETT is preferred over an LMA to accommodate higher airway pressures (larger tidal volumes and increased PEEP). LMA use may increase the risk of gastric insufflation with higher pressures. This in turn may increase the risk for aspiration, although the risk remains low based on previous studies [48]. Following bronchoscopy, the ETT can be exchanged for an LMA to facilitate complete EBUS staging if desired. LMA may be feasible in smaller patients (e.g., < 80 kg) provided that a good seal can be obtained whilst providing the recommended tidal volumes and PEEP. Jet ventilation has also been used to minimize motion and CT-to-body divergence [35, 49], but may be limited by availability and expertise.

General anesthesia using total intravenous anesthesia (TIVA) with propofol and muscle paralysis is optimal [47]. Lengthy intubation times may increase the risk of atelectasis, thus, avoid “traditional” rapid-sequence intubation (i.e., avoid the use of FiO_2_ of 1.0). Instead, perform an expeditious intubation using non-depolarizing muscle relaxants rather than suxamethonium. Application of PEEP throughout induction has also been shown to prevent atelectasis [42]. Whilst the use of volatile anesthetics is not contraindicated, repeated opening of the circuit for the passage of the scope poses additional challenges to maintaining the depth of anesthesia and volatile gas pollution in the operating room.

### Recruitment maneuvers

**Fig. 2:**
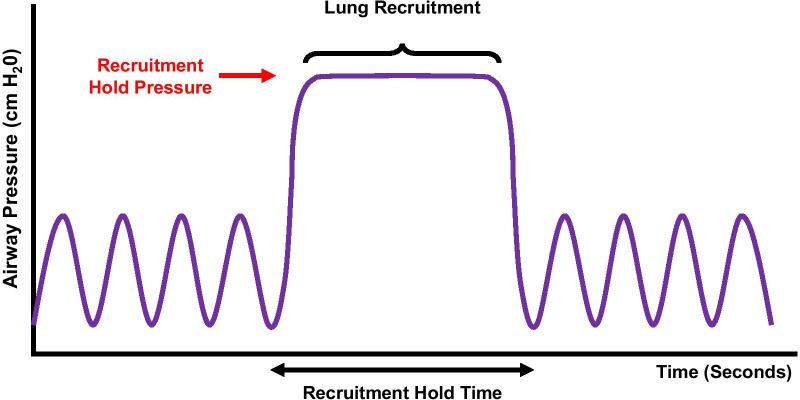
Recruitment maneuvers after intubation may reverse any induction-related atelectasis and assess hemodynamic stability during higher PEEP. Hemodynamic instability may limit use of the traditional ‘40 for 40’ hold. Higher PEEP for shorter duration may be considered

Reducing the FiO_2_ to the lowest tolerable percentage after intubation will improve airway visualization. Unless contraindicated (e.g., due to acute respiratory distress syndrome, ventilator-induced lung injury, recent segmentectomy, structural lung disease, or surgery), conduct up to four recruitment maneuvers immediately after intubation [46] to reverse any intubation atelectasis and to assess hemodynamic stability during higher PEEP (Fig. 2). This is especially important if intubation was prolonged due to a difficult intubation. Hemodynamic instability may preclude the use of traditional recruitment maneuvers, such as the use of 40 cm H_2_O for 40 s (the ‘40 for 40 hold’). Judicious use of PEEP from the pre-induction phase and throughout the procedure is recommended [21, 38, 42], but may vary based on the patient’s hemodynamic tolerance. Maintain higher PEEP with the lowest tolerable FiO_2_ as guided by oxygen saturation. PEEP of up to 10–12 cm H_2_O may be beneficial for upper lobe biopsies, and even higher PEEP may be required for lower lobe biopsies (particularly in obese patients) due to the lower functional residual capacity and increased risk of atelectasis in the dependent areas. An increase in tidal volumes may be considered if tolerated.

**Fig. 3:**
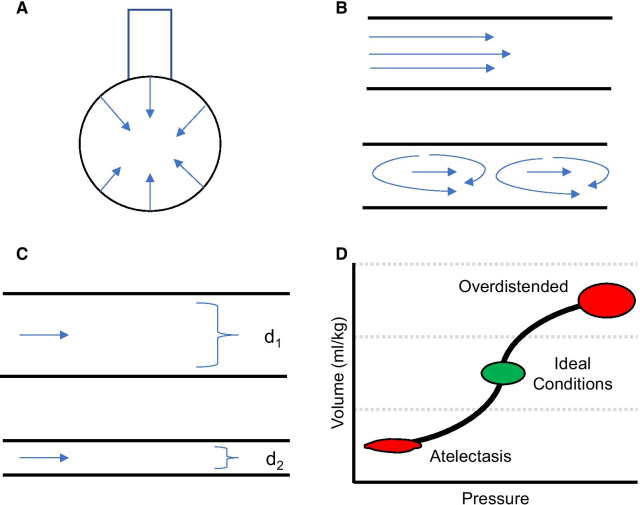
Mechanisms leading to atelectasis during bronchoscopy and increased airway resistance. **A** Surface tension caused by water molecules leads to attraction. **B** Once the airflow is disrupted in atelectatic lung units, turbulent airflow leads to increased airway resistance. **C** As the bronchi decrease in diameter, there is a substantial increase in airways resistance. **D** Compliance curve for alveoli. As the lung deflates, resistance increases and gas flow through the airways decreases. Atelectatic lung units have poor compliance and require significant changes in inflation pressure to result in minor changes in volume

Use of higher PEEP and tidal volumes will help maintain optimal lung inflation [44]. However, use of these higher settings should be guided by what the patient is able to tolerate hemodynamically, as demonstrated in the post-intubation recruitment maneuvers. This is especially important in obese patients or those with structural lung disease who may be at increased risk of barotrauma or volutrauma. Also, the risk of barotrauma is minimized when the sheer stress of repeated alveolar re-expansion is avoided, and the alveoli are held on a favorable part of the compliance curve (Fig. 3). While intuitively contradictory to the classical teaching of low tidal volume to prevent barotrauma, airway visualization, accurate lesion localization, and successful biopsy require these preprocedural conditions to be replicated as closely as possible.

### Breath-hold for image acquisition

#### Timing the breath-hold

**Fig. 4:**
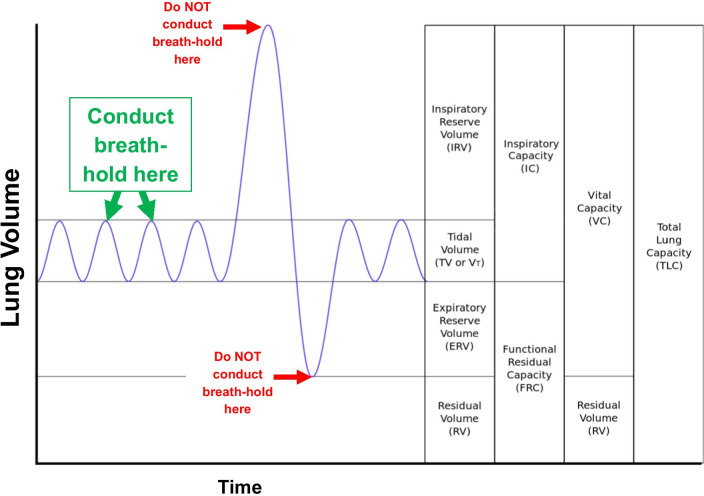
A breath-hold is required to reduce motion artifact during intraprocedural imaging (e.g., CBCT, digital tomosynthesis). The breath-hold should be performed at peak inspiration, not at end-expiration. This does not require a vital capacity maneuver but should occur at the end of a normal tidal breath. Adapted from Kapwatt at English Wikipedia (https://commons.wikimedia.org/w/index.php?curid=74891988) and used with permission under the terms of Creative Commons License CC BY-SA 3.0 ()

A breath-hold is required to reduce motion artifact and provide clearer, more accurate images during certain image-guided bronchoscopy procedures (e.g., fluoroscopic navigation, CBCT). These procedures require a complete lack of movement beyond cardiac pulsations; therefore, a carefully timed breath-hold is essential. The breath-hold should be performed at peak inspiration, not at end-expiration. This does not require a vital capacity maneuver, rather, it should occur at the end of a normal inspiratory breath (Fig. 4).

#### Maintaining a constant circuit pressure and PEEP

The breath-hold may be achieved by either the anesthesia machine’s automatic feature or manually. The automatic feature sets and maintains the appropriate pressure using the Vital Capacity Hold Function. In the manual method, the breath-hold is initiated by switching the ventilator to manual mode. Adjusting the adjustable pressure-limiting (APL) valve during breath hold maneuvers is required to maintain a constant circuit pressure and PEEP and reduce diaphragmatic movement once a leak is created in the circuit with the passage of the bronchoscope. The measured circuit pressure reflects intrapulmonary pressure (PEEP) and is a balance between fresh gas inflow and leak from the circuit plus outflow via the APL. Therefore, it’s preferable to focus on the circuit pressure reading (shown either on the digital screen or on the circuit pressure manometer) and adjust the APL manually to maintain this pressure at the designated PEEP, rather than on an absolute APL number. Vigilance is required to ensure that there is a balance of fresh gas inflow to circuit leak. These measures will minimize motion artifact of the diaphragm during imaging which can cause blurring and thereby hinder image interpretation by the clinician.

#### Maintaining the breath-hold

**Fig. 5:**
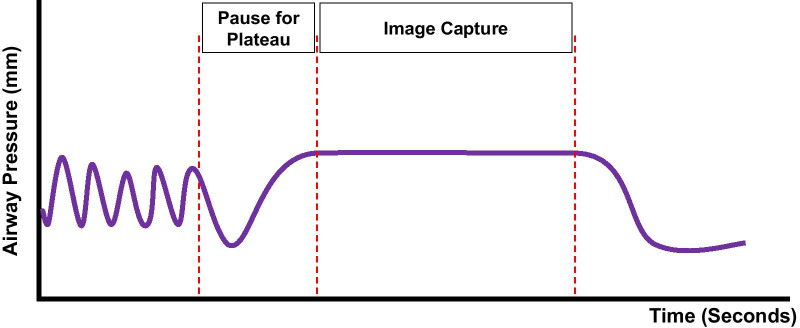
To prevent motion artifact, maintain the breath-hold until pressures plateau before beginning the imaging sweep (5–10 s). Be aware of the potential for hemodynamic changes due to prolonged breath-hold

The breath-hold should be maintained for a sufficient time to allow pressures to plateau and equilibrate throughout the bronchial tree prior to beginning intraprocedural imaging (Fig. 5). The proceduralist should wait 5–10 s before beginning the imaging sweep after the anesthesiologist initiates the breath-hold (once PEEP is stabilized as measured by constant circuit pressure). This gives time for the intrapulmonary pressures to equilibrate before the imaging sweep begins. These measures will remove motion artifact, provide clearer images, and allow for more accurate localization of the lung lesion. By maintaining high PEEP, the bronchial tree is also optimally dilated. The imaging sweep will add additional time to the breath-hold (up to 30 s, depending on the system used). Therefore, it is important to be aware of the potential for hemodynamic changes due to prolonged breath-hold during the equilibration and imaging sweep.

### Biopsy procedure

The recommended anesthesia settings described above should be maintained throughout the biopsy procedure to ensure that lung volumes are consistent with those present during intraprocedural imaging. Any changes during the biopsy procedure would necessitate a repeat of both the breath-hold and the imaging sweep.

### Post-procedure

Routine reversal and standard assessment of fitness for extubation should be employed, as should suctioning of secretions. Standard post-procedural discharge criteria should be employed, and a postprocedure chest x-ray is recommended to exclude complications such as pneumothorax. Patients should be completely reversed from neuromuscular paralysis.

## Clinical evidence

The proposed ventilation protocol has been evaluated in a retrospective, single-center study of subjects with peripheral lung lesions < 30 mm undergoing CBCT with augmented fluoroscopy [28]. In two non-randomized, consecutively enrolled groups (25 subjects per group), a conventional, non-standardized ventilation protocol (typically 100% FiO_2_ and PEEP set to 0 or 5 cm H_2_O) was compared to the optimized ventilation protocol. Two independent reviewers observed significantly less atelectasis in dependent areas (*P* = 0.0001) and in sublobar or lobar regions (*P* = 0.01) using the optimized protocol. There was also a significantly smaller proportion of lesions obscured by atelectasis using the optimized protocol (8% Reviewer 1, 4% Reviewer 2) than the conventional protocol (36% observed by both reviewers). Furthermore, this significant reduction in atelectasis was accompanied by a higher diagnostic yield, though not statistically significant (70% conventional versus 92% optimized, *P* = 0.08). Pneumothorax not requiring a chest tube was observed in 1 patient following the optimized protocol compared to 0 patients with conventional ventilation (*P* = 1.0). A study of tomosynthesis-based fluoroscopic navigation used a similar ventilation protocol, with intubation under TIVA, neuromuscular blockade, a recruitment maneuver after intubation, minimized oxygen, and 15 cm H_2_O applied throughout the procedure. A diagnostic yield of 77% was observed, with a pneumothorax rate of only 2.5% (8/324) [10]. While future randomized studies are necessary to confirm these results, this data suggests that a ventilation protocol optimized for advanced guided bronchoscopy not only reduces atelectasis, but may also increase in diagnostic yield. The randomized VESPA trial is (NCT04311723) currently enrolling and aims to compare a ventilation strategy designed to prevent atelectasis during bronchoscopy compared to conventional mechanical ventilation [50].

## Conclusions

Atelectasis is common during advanced guided bronchoscopic biopsy [25] and can cause CT-to-body divergence [8], interfering with the ability to obtain diagnostic tissue [28]. The anesthesia techniques proposed in this paper are designed to stabilize dependent areas of the lung, reduce atelectasis, minimize motion artifact, and provide more accurate localization during guided bronchoscopy. Future randomized studies are needed to prospectively compare bronchoscopy procedures with and without optimized ventilation strategies.

## Supplementary Information

**Additional file 1.** Literature search methods.

## Data Availability

Not applicable.

## Abbreviations

APL: Adjustable pressure-limiting (valve)
ASA: American Society of Anesthesiologists
CBCT: Cone-beam computed tomography
CT: Computed tomography
EBUS: Endobronchial ultrasound
ETT: Endotracheal tube
FiO_2_: Fraction of inspired oxygen
LMA: Laryngeal mask airway
PEEP: Positive end-expiratory pressure
TIVA: Total intravenous anesthesia
