# Supplementary material for: Anesthesia considerations to reduce motion and atelectasis during advanced guided bronchoscopy
Source: BMC Pulm Med. 2021 Jul 17;21:240. doi: 10.1186/s12890-021-01584-6 (PMC8286573; doi:10.1186/s12890-021-01584-6)
Supplement: Supplementary file 1 — Additional file 1. Literature search methods. [file 12890_2021_1584_MOESM1_ESM.docx]

**Additional File 1: Literature Search Methods**

**Article:** Anesthesia considerations to reduce motion and atelectasis during advanced guided bronchoscopy

**Authors**: Michael A. Pritchett DO MPH, Kelvin Lau MA BMBCh(Oxon) DPhil(Oxon) FRCS(CTh), Scott Skibo MD, Karen A. Phillips MBChB FCA MBA, Krish Bhadra MD

**Literature Search Methods**

A systematic literature search was conducted for papers evaluating the impact of anesthesia methods on outcomes during advanced peripheral bronchoscopy (including electromagnetic navigation bronchoscopy, radial endobronchial ultrasound, virtual bronchoscopic navigation, cone-beam computed tomography, augmented fluoroscopy, and robotic bronchoscopy).

The search was conducted in two parts. First, a title/abstract search was conducted to capture studies that specifically evaluated the impact of the anesthesia/sedation method on outcomes of advanced peripheral bronchoscopic biopsy (see **Table 1** below). Second, to capture multicenter studies and meta-analyses that compared anesthesia methods as part of multivariate analyses, a second broader full-text search was conducted (see **Table 2** below). PubMed/MEDLINE and Embase were searched for English-language articles published from 2005 through May 24, 2021 (Search #1) or May 26, 2021 (Search #2).

A total of 154 peer-reviewed journal articles were identified and screened, of which 116 were excluded based on title and abstract review (see **Figure 1**). After full-text review of the remaining 38 papers, there were 11 papers that specifically evaluated the impact of the anesthesia/sedation method on atelectasis, safety, or diagnostic yield during advanced peripheral guided bronchoscopy. These included 8 original studies [1-8] and 3 meta-analyses [9-11].


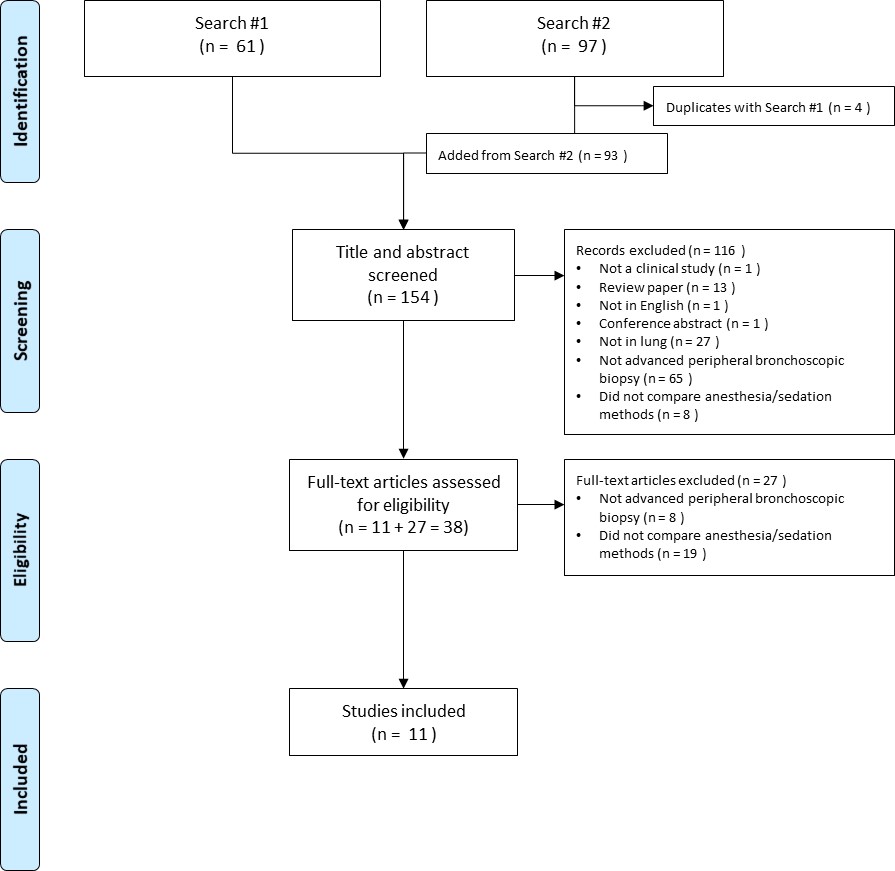


**Figure 1. Number of Articles Assessed**

| **Table 1.** Search #1 Strategy | | |
| --- | --- | --- |
| **Database(s):** **Journals@Ovid Full Text**May 25, 2021**, Embase**1974 to 2021 May 24**, Ovid MEDLINE(R) ALL**1946 to May 24, 2021 | | |
| **#** | **Searches** | **Results** |
| 1 | (((bronch* or endobronch* or hemithora* or hemi thora* or intrabronch* or intrapulmonar* or lobe* or lung* or pulmonar*) adj3 (cancer* or carcino* or lesion* or malignan* or metastas* or metastati* or neoplas* or neo plas* or tumor* or tumour*) adj6 (aspirat* or biops* or needl* or fineneedl* or (specimen* adj2 (retriev* or gather* or sampl*)))) and (transbronch* or trans bronch* or transthorac* or trans thorac* or electromagnetic navigational bronchoscop* or enb or superdimension*)).ti,ab,kw,sh,hw. | 6093 |
| 2 | ((transbronch* or trans bronch* or transthorac* or trans thorac* or electromagnetic navigational bronchoscop* or enb or superdimension*) adj6 (guid* or aspirat* or biops* or needl* or fineneedl* or diagnos* or identifi* or recogni* or discover* or detect* or determin* or confirm* or verificat*)).ti,ab,kw,sh,hw. | 48778 |
| 3 | ((bronch* or endobronch* or hemithora* or hemi thora* or intrabronch* or intrapulmonar* or lobe* or lung* or pulmonar*) adj3 (cancer* or carcino* or lesion* or malignan* or metastas* or metastati* or neoplas* or neo plas* or tumor* or tumour*)).ti,ab,kw,dm,dv,tn,mf,kf,hw,sh,fx,ec,xt,tx. | 1361660 |
| 4 | 1 or (2 and 3) | 16410 |
| 5 | ((Auris* adj4 Monarch*) or ((Body Vision* or BodyVision*) adj4 (LungVision* or Lung Vision*)) or (Broncus* adj3 Archimedes*) or ((ION or IONR or IONReg* or IONTM or IONTrade*) adj4 Intuitiv*) or (robot* adj4 bronchoscop*) or ((augment* or navigat* or tomosynth*) adj3 fluoroscop*)).ti,ab,kw,dm,dv,tn,mf,kf,hw,sh,fx,ec,xt,tx. | 2996 |
| 6 | 4 or 5 | 19255 |
| 7 | (anesthe* or anaesthe* or sedat* or ventilat*).ti. | 560048 |
| 8 | 6 and 7 | 128 |
| 9 | (conference* or congress* or meeting* or poster* or symposia* or symposium* or (oral* and (presentation* or session*)) or (scientific* and session*) or work shop* or workshop*).dt,pt. or (abstract* or poster or posters).dt,pt,ti. or (editorial* or letter or note or book or patent* or comment or comments or interview or interviews).dt,pt,ti. or (trade journal* or audio* or video*).dt,pt. | 13116381 |
| 10 | ((animal or animals or avian* or beagle* or bovine* or bull or bulls* or cadaver* or calf or calve* or canine* or cat or cats or cattle or cow* or dog or dogs or equine* or feline* or ferret* or fish* or foal* or frog* or gerbil* or goat* or hamster* or horse* or invertebrate* or lamb* or mammal* or mare* or marmoset* or mice or minipig* or mini pig* or monkey* or mouse* or murine* or ovine* or pig or piglet* or pigs or porcine* or primate* or rabbit* or rat or rats or rodent* or sheep* or swine* or veterinar*) adj5 (experiment* or investigat* or methods or model* or randomi* or research or studies or study or subjects or trial or trials)).ti,ab,dt,pt,ct,kf,hw,sh. or (animal* or veterinar* or pet medicine*).so,jn,jx,jw. | 6918124 |
| 11 | 8 not (9 or 10) | 93 |
| 12 | limit 11 to yr="2005 -Current" | 83 |
| 13 | remove duplicates from 12 | 61 |

| ***Table 2.*** *Search #2 Strategy* | | |
| --- | --- | --- |
| **Database(s):** **Journals@Ovid Full Text**May 27, 2021**, Embase**1974 to 2021 May 26**, Ovid MEDLINE(R) ALL**1946 to May 26, 2021 | | |
| **#** | **Searches** | **Results** |
| 1 | (((bronch* or endobronch* or hemithora* or hemi thora* or intrabronch* or intrapulmonar* or lobe* or lung* or pulmonar*) adj3 (cancer* or carcino* or lesion* or malignan* or metastas* or metastati* or neoplas* or neo plas* or tumor* or tumour*) adj6 (aspirat* or biops* or needl* or fineneedl* or (specimen* adj2 (retriev* or gather* or sampl*)))) and (transbronch* or trans bronch* or transthorac* or trans thorac* or electromagnetic navigational bronchoscop* or enb or superdimension*)).ti,ab,kw,sh,hw. | 6097 |
| 2 | ((transbronch* or trans bronch* or transthorac* or trans thorac* or electromagnetic navigational bronchoscop* or enb or superdimension*) adj6 (guid* or aspirat* or biops* or needl* or fineneedl* or diagnos* or identifi* or recogni* or discover* or detect* or determin* or confirm* or verificat*)).ti,ab,kw,sh,hw. | 48793 |
| 3 | ((bronch* or endobronch* or hemithora* or hemi thora* or intrabronch* or intrapulmonar* or lobe* or lung* or pulmonar*) adj3 (cancer* or carcino* or lesion* or malignan* or metastas* or metastati* or neoplas* or neo plas* or tumor* or tumour*)).ti,ab,kw,dm,dv,tn,mf,kf,hw,sh,fx,ec,xt,tx. | 1362092 |
| 4 | ((Auris* adj4 Monarch*) or ((Body Vision* or BodyVision*) adj4 (LungVision* or Lung Vision*)) or (Broncus* adj3 Archimedes*) or ((ION or IONR or IONReg* or IONTM or IONTrade*) adj4 Intuitiv*) or (robot* adj4 bronchoscop*) or ((augment* or navigat* or tomosynth*) adj3 fluoroscop*)).ti,ab,kw,dm,dv,tn,mf,kf,hw,sh,fx,ec,xt,tx. | 2996 |
| 5 | (((clinical* or comparison* or comparative* or control* or multicent* or multi cent* or multiinstitution* or multinstitution* or multi institution* or single center* or single centre* or randomiz* or randomis*) adj4 (analys* or analyz* or investigation or investigations or review* or study or studies or trial or trials)) or guideline* or guide line* or metaanaly* or metanaly* or meta analy* or registries or (systematic* adj4 review*)).ti,ab,dt,pt. | 7107868 |
| 6 | (bivariat* or bi variat* or multivariat* or multi variat*).ti,ab,kw,dm,dv,tn,mf,kf,hw,sh,fx,ec,xt,tx. | 1575707 |
| 7 | (conference* or congress* or meeting* or poster* or symposia* or symposium* or (oral* and (presentation* or session*)) or (scientific* and session*) or work shop* or workshop*).dt,pt. or (abstract* or poster or posters).dt,pt,ti. or (editorial* or letter or note or book or patent* or comment or comments or interview or interviews).dt,pt,ti. or (trade journal* or audio* or video*).dt,pt. | 13119443 |
| 8 | ((animal or animals or avian* or beagle* or bovine* or bull or bulls* or cadaver* or calf or calve* or canine* or cat or cats or cattle or cow* or dog or dogs or equine* or feline* or ferret* or fish* or foal* or frog* or gerbil* or goat* or hamster* or horse* or invertebrate* or lamb* or mammal* or mare* or marmoset* or mice or minipig* or mini pig* or monkey* or mouse* or murine* or ovine* or pig or piglet* or pigs or porcine* or primate* or rabbit* or rat or rats or rodent* or sheep* or swine* or veterinar*) adj5 (experiment* or investigat* or methods or model* or randomi* or research or studies or study or subjects or trial or trials)).ti,ab,dt,pt,ct,kf,hw,sh. or (animal* or veterinar* or pet medicine*).so,jn,jx,jw. | 6920070 |
| 9 | ((1 or (2 and 3) or 4) and 5 and 6) not (7 or 8) | 152 |
| 10 | limit 9 to yr="2005 -Current" | 146 |
| 11 | remove duplicates from 10 | 97 |

**Articles Included**

1. Bhadra K, Setser RM, Condra W, Pritchett MA. Lung Navigation Ventilation Protocol to Optimize Biopsy of Peripheral Lung Lesions. J Bronchology Interv Pulmonol. 2021;doi: 10.1097/LBR.0000000000000756.

2. Bowling MR, Kohan MW, Walker P, Efird J, Ben Or S. The effect of general anesthesia versus intravenous sedation on diagnostic yield and success in electromagnetic navigation bronchoscopy. J Bronchology Interv Pulmonol. 2015;22:5-13.

3. Folch EE, Pritchett MA, Nead MA, Bowling MR, Murgu SD, Krimsky WS, et al. Electromagnetic Navigation Bronchoscopy for Peripheral Pulmonary Lesions: One-Year Results of the Prospective, Multicenter NAVIGATE Study. J Thorac Oncol. 2019;14:445-458.

4. Minami D, Nakasuka T, Ando C, Iwamoto Md Y, Sato K, Fujiwara K, et al. Bronchoscopic diagnosis of peripheral pulmonary lung cancer employing sedation with fentanyl and midazolam. Respir Investig. 2017;55:314-317.

5. Ost DE, Ernst A, Lei X, Kovitz KL, Benzaquen S, Diaz-Mendoza J, et al. Diagnostic Yield and Complications of Bronchoscopy for Peripheral Lung Lesions. Results of the AQuIRE Registry. Am J Respir Crit Care Med. 2016;193:68-77.

6. Tanner NT, Yarmus L, Chen A, Wang Memoli J, Mehta HJ, Pastis NJ, et al. Standard Bronchoscopy With Fluoroscopy vs Thin Bronchoscopy and Radial Endobronchial Ultrasound for Biopsy of Pulmonary Lesions: A Multicenter, Prospective, Randomized Trial. Chest. 2018;154:1035-1043.

7. Towe CW, Nead MA, Rickman OB, Folch EE, Khandhar SJ, Perry Y, et al. Safety of Electromagnetic Navigation Bronchoscopy in Patients With COPD: Results From the NAVIGATE Study. J Bronchology Interv Pulmonol. 2019;26:33-40.

8. Webb TN, Bonta DV, Masters R, Parks C, Bechara R. Jet Ventilation Decreases Target Motion and Increases Yield of ENB Especially in Lesions With Negative Bronchus Sign. J Bronchology Interv Pulmonol. 2020;27:14-21.

9. Folch EE, Labarca G, Ospina-Delgado D, Kheir F, Majid A, Khandhar SJ, et al. Sensitivity and Safety of Electromagnetic Navigation Bronchoscopy for Lung Cancer Diagnosis: Systematic Review and Meta-analysis. Chest. 2020;158:1753-1769.

10. Gex G, Pralong JA, Combescure C, Seijo L, Rochat T, Soccal PM. Diagnostic yield and safety of electromagnetic navigation bronchoscopy for lung nodules: a systematic review and meta-analysis. Respiration. 2014;87:165-176.

11. Zhang W, Chen S, Dong X, Lei P. Meta-analysis of the diagnostic yield and safety of electromagnetic navigation bronchoscopy for lung nodules. J Thorac Dis. 2015;7:799-809.
